# Dissecting the genetic architecture of sunflower disc diameter using genome‐wide association study

**DOI:** 10.1002/pld3.70010

**Published:** 2024-10-09

**Authors:** Yavuz Delen, Ravi V. Mural, Semra Palali‐Delen, Gen Xu, James C. Schnable, Ismail Dweikat, Jinliang Yang

**Affiliations:** ^1^ Department of Agronomy and Horticulture University of Nebraska‐Lincoln Lincoln NE USA; ^2^ Department of Agronomy, Horticulture and Plant Science South Dakota State University Brookings SD USA; ^3^ Center for Plant Science Innovation University of Nebraska‐Lincoln Lincoln NE USA

**Keywords:** disc diameter, GWAS, helianthus, sunflower

## Abstract

Sunflower ( *Helianthus annuus* L.) plays an essential role in meeting the demand for edible oil worldwide. The yield of sunflower seeds encompasses several component traits, including the disc diameter. Over three consecutive years, 2019, 2020, and 2022, we assessed phenotypic variation in disc diameter across a diverse set of sunflower accessions (N = 342) in replicated field trials. Upon aggregating the phenotypic data from multiple years, we estimated the broad sense heritability (*H* ^2^) of the disc diameter trait to be 0.88. A subset of N = 274 accessions was genotyped by using the tunable genotyping‐by‐sequencing (tGBS) method, resulting in 226,779 high‐quality SNPs. Using these SNPs and the disc diameter phenotype, we conducted a genome‐wide association study (GWAS) employing two statistical approaches: the mixed linear model (MLM) and the fixed and random model circulating probability unification (farmCPU). The MLM and farmCPU GWAS approaches identified 106 and 8 significant SNPs located close to 53 and 21 genes, respectively. The MLM analysis identified two significant peaks: a prominent signal on chromosome 10 and a relatively weaker signal on chromosome 16, both of which were also detected by farmCPU. The genetic loci associated with disc diameter, as well as the related candidate genes, present promising avenues for further functional validation and serve as a basis for sunflower oil yield improvement.

## 1 INTRODUCTION

Sunflower (*Helianthus annuus* L.), a member of the *Helianthus* genus from the Compositae (Asteraceae) family, is a diploid crop with 2n = 34 chromosomes. It is native to North America and was initially domesticated by Native Americans in the East Central region of the US (Blackman et al., 2011). Sunflower is the second‐largest hybrid crop after maize (*Zea mays* L.) (Seiler et al., 2017) and the fourth‐largest crop in oil production after palm, soybean, and rapeseed (FAO, 2021), providing 25–50% edible oil in seeds (Khoufi et al., 2014). Most sunflower hybrids contain 45–50% oil in their seeds (Jocic et al., 2015). The ability of sunflower populations to adapt to a wide range of environments has resulted in their cultivation around the globe on every continent except Antarctica (Seiler et al., 2017). They are cultivated in more than 70 countries worldwide (Rauf et al., 2012), providing more than 13% of the total edible oil globally (Rauf et al., 2008). A wide range of phenotypic variation in terms of the desired traits (i.e., seed size, disc diameter, oil content, resistance to biotic and abiotic factors) and the genetic diversity in the domesticated and wild sunflower populations provide a valuable source for sunflower breeding programs and genetic studies. Sunflower's ability to grow on disrupted and marginal agricultural land suggests it may play an increasingly crucial role in meeting the needs of the increasing world population.

Sunflowers are mostly grown for seed production to extract oil from the seeds or for confectionery purposes. The sunflower seed yield is affected by various traits like seed size and seed weight. In addition to those, floral traits play an essential role in seed yield. As a floral trait, the disc diameter trait of the sunflower is one of the most important traits. The sunflower discs consist of up to 3,000 tubular disc flowers, which are fertile and produce the seed known as cypsela or achene (Marek, 2019). The non‐oilseed hybrids with up to 8,000 disc flowers are also available (Harveson et al., 2016). It has been indicated that disc size and the number of seeds are highly correlated with each other (Alkio et al., 2003; Marinković, 1992; Palmer & Steer, 1985), contributing directly to sunflower seed yield (Machikowa & Saetang, 2008; Yasin & Singh, 2010). A high variation in disc size is present in the sunflower populations. In contrast to wild progenitors, domesticated sunflowers have wider discs (Balalić et al., 2016; Onemli & Gucer, 2010; Radanović et al., 2018). Studies show that sunflower disc diameter is a quantitative trait affected by many genetic loci, including both additive and non‐additive genetic components (Abdelsatar et al., 2020; Gvozdenović et al., 2005; Hladni et al., 2014).

Sunflower has a relatively La genome size (3.6 Gb), and its genome assembly that is used as a reference genome was released in 2017 (Badouin et al., 2017). With technological advances, cost‐effective and easily reachable genotyping opened new perspectives to plant breeding and genetics programs by enabling genome‐wide studies. To assess the genetic mechanism controlling sunflower traits, many quantitative trait loci (QTL) and genome‐wide association studies (GWAS) have been conducted (Badouin et al., 2017; Chernova et al., 2021; Dowell et al., 2019; Fang, 2017; Goryunov et al., 2019; Hasson et al., 2021; Mangin et al., 2017; Masalia et al., 2018; Reinert et al., 2020; Talukder et al., 2014), resulting in hundreds of trait‐associated loci. Among those GWAS studies, only Dowell et al. (2019) focused on the disc diameter trait, used about 6,000 markers to detect association signals for the disc size, and found trait‐associated loci in large genomic regions. Higher‐resolution GWAS, however, is needed to further dissect the genetic components in determining the disc diameter traits.

This study aims to reveal the genetic basis controlling sunflower disc diameter variation using a set of high‐density SNP markers. For this purpose, we genotyped a sunflower diversity panel using tGBS methods and obtained 226,779 high‐quality SNPs. Meanwhile, we collected the phenotypic data of diverse sunflower accessions in the years 2019, 2020, and 2022 in the replicated field trials. Subsequently, we conducted GWAS and identified 106 and 8 significant SNPs using the MLM and farmCPU methods, respectively. These trait‐associated SNPs and the candidate genes underneath the GWAS peaks can be potentially applied for further sunflower improvement.

## 2 MATERIALS AND METHODS

### 2.1 Plant materials and field experimental design

A set of 342 sunflower accessions, originally collected in 25 different countries on six continents, were obtained from the North Central Regional Introduction Station (NCRPIS) in Ames, Iowa, USA. Accessions originating from diverse geographical locations were randomly selected to maximize genetic diversity.

These accessions were grown at the University of Nebraska‐Lincoln's research farm at Havelock in 2019 (40^*°*^ 51^*′*^ 15.9^*′′*^ *N*, 96^*°*^ 36^*′*^ 42.6^*′′*^ *W*), 2020 (40^*°*^ 51^*′*^ 26.5^*′′*^ *N*, 96^*°*^ 36^*′*^ 53.4^*′′*^ *W*), and 2022 (40^*°*^ 51^*′*^ 20.1^*′′*^ *N*, 96^*°*^ 36^*′*^ 32.4^*′′*^ *W*). For the field experiment, an incomplete block design was employed with two main blocks, four split plots per block, and three replicates of the check (PI 432513) per split‐plot. Each genotype was planted in a 3.6‐m‐long single row with .75‐m row spacing and an alleyway of .9 m. Twelve seeds were planted per row, leading to a density of about 45,000 plants per hectare. The disc diameters (in centimeters) were measured for three representative plants per plot, excluding edge plants. Measurements were carried out after pollination but prior to maturity.

### 2.2 Phenotypic data processing and heritability calculation

Best Linear Unbiased Prediction (BLUP) calculation for the disc diameter was calculated using the lme4 package (Bates et al., 2015) in R (v 4.2.0) (R Core Team, 2020). In the analysis, the following model was fit using phenotype data collected in 2019, 2020, and 2022: *Y ∼* (1*|genotype*) + (1*|block*) + (1*|split − plot*) + (1*|year*) + (1*|genotype*: *year*) + *error*, where *Y* represents the phenotype (Sunflower disc diameter). In this study, genotype, block, split‐plot, year, and genotype‐by‐year interaction were treated as random effects. The BLUP calculated using the combination of data collected in 2019, 2020, and 2022 was used in the GWAS analysis.

In the BLUP model,

$$y_{\text{ijkrl}}=\mu +g_{i}+t_{l}+g_{i}*t_{l}+b_{\mathrm{jrl}}+s_{\text{jkrl}}+q_{\mathrm{rl}}+\varepsilon$$

where *y*_*i jkrl*_ refers to the phenotypic value of the *i* ^*th*^ genotype evaluated in the *k* ^*th*^ split‐plot of the *j* ^*th*^ block of the *r* ^*th*^ replicate nested within the *l* ^*th*^ year; *μ* is the overall mean; *g*_*i*_ is the random effect of the *i* ^*th*^ genotype; *t*_*l*_ is the random effect of the *l* ^*th*^ year; *g*_*i*_ *t_*l*_ is the random effect of the *i* ^*th*^ genotype with the *l* ^*th*^ year interaction; *b*_*jrl*_ is the random effect of the *j* ^*th*^ block of the *r* ^*th*^ replicate within the *l* ^*th*^ year; *s*_*jkrl*_ is the random effect of the *k* ^*th*^ split‐plot of the *j* ^*th*^ block of the *r* ^*th*^ replicate within the *l* ^*th*^ year; *q*_*rl*_ is the random effect of the *r* ^*th*^ replicate nested within the *l* ^*th*^ year; *ε* is the random residual error.

Broad‐sense heritability (*H* ^2^) was calculated based on the equation *H* ^2^ = *V*_*G*_ */V*_*P*_ (Falconer & Mackay, 1996; Xu, 2022), where *V*_*P*_ is *V*_*G*_ + *V*_*E*_, *V*_*G*_ is total genetic variance, *V*_*P*_ is total phenotypic variance, and *V*_*E*_ is phenotypic variance due to environmental factors. Regarding this, the broad‐sense heritability of sunflower disc size was calculated for the combined environments of 2019, 2020, and 2022 by the following equation

$$H^{2}=\frac{\sigma_{g}^{2}}{\sigma_{g}^{2}+\frac{\sigma_{\mathrm{gxy}}^{2}}{n}+\frac{\sigma^{2}e}{\mathrm{nr}}}$$

where *σ* ^*2*^_*g*_ is the components of variance for genotype, *σ* ^*2*^_*e*_ is the components of variance for the environment, *σ* ^*2*^_*gxy*_ is the components of variance for genotype by year interaction, *n* is the number of years, and *r* is replications.

### 2.3 DNA extraction and genotyping

All accessions were grown in a greenhouse to collect leaf samples for DNA extraction. The accessions were grown on 10x10 cm pots using a standard greenhouse mix with a ratio of 5 gal of peat, 3 gal of soil, 2.5 gal of sand, and 2.5 gal of vermiculite. Two weeks after planting, samples (600–700 mg) from young leaves at the V4 stage were collected in sterile Eppendorf tubes, and then the samples were placed in a *−* 80^*°*^ Celsius freezer. Frozen samples were lyophilized using a freeze‐drying machine (VirTis 25XL) for three days at *−*70^*°*^ Celsius. The lyophilized leaf samples were transferred into the tubes of three 96‐well collection kits and shipped to Freedom Markers in Ames, Iowa (https://www.freedommarkers.com/) for genotyping. The accession “PI 490282” was placed once in each well plate as a check that can be used to confirm the quality of genotyping. The company used the BioSprint MagAttract 96 DNA Plant Core Kit by QIAGEN for DNA extraction and utilized a PicoGreen kit on an Eppendorf Plate Reader for assessing purity and quality control. The SNP genotyping was conducted by Freedom Markers using tGBS® genotyping by sequencing technology utilizing restriction enzyme Bsp1286I (Ott et al., 2017). The tGBS libraries were constructed using the QCd DNA (as described above) from each sample, and the resulting libraries were subsequently sequenced on an Illumina HiSeq X with 2 *×* 150 bp paired‐end reads. Due to the high missing rate, 11 of the 285 samples (PI_219650, PI_262519, PI_262521, PI_432522, PI_531502, PI_546360, PI_552946, PI_599770, PI_599781, PI_632338, and PI_650658) that were selected for genotyping were removed in subsequent analyses.

### 2.4 Sequence data processing and SNP calling

The resulting fastq sequence data was scanned and quality‐trimmed by Freedom Markers using internal software that removes low‐quality regions in a two‐step process. In the first step, reads were scanned beginning at each end. If the nucleotide had PHRED quality values less than 15 (Ewing et al., 1998; Ewing & Green, 1998) they were discarded. In the second step, the read was scanned in 10 base pair windows and truncated if the average PHRED quality score of a 10 base pair window fell below the cutoff value of 15. These trimming parameters were adapted from the trimming software “LUCY2” (Chou & Holmes, 2001; Li & Chou, 2004).

Reads were aligned to the HanXRQr2.0‐SUNRISE sunflower reference genome (https://www.ncbi.nlm.nih.gov/assembly/GCF_002127325.2/) (Badouin et al., 2017) using GSNAP (Wu & Nacu, 2010). Only confidently and uniquely mapped reads, defined as (*≤* 2 mismatches every 36 bp and less than 5 bases for every 75 bp as tails) were used for subsequent analyses. SAMtools was used for the conversion of alignment file formats (http://samtools.sourceforge.net/) (Li et al., 2009). Polymorphic sites that diverge from the reference genome in at least one sample were identified by utilizing all reads that uniquely align with the sunflower reference genome. After counting the reads at each SNP site that was considered to have potential, it was checked if the SNP site was interrogated, meaning that a SNP site was supported by at least five unique reads. Freedom Markers reported the number of positions in the genome. If a segregating polymorphism existed at a certain location in the genome, a SNP marker was detected and genotyped.

A SNP was genotyped as homozygous in a given individual when at least 80% of all aligned reads at that site supported the most commonly observed nucleotide, and at least five unique reads must support the most commonly observed nucleotide. For an individual to be genotyped as heterozygous at a given site at least 30% of all aligned reads that cover that position and at least five unique reads must support each of the two most commonly observed nucleotides, and at least 80% of all aligned reads covering that nucleotide site must support one of those two most commonly observed nucleotides. In both homozygous and heterozygous SNP criteria, polymorphisms in the first and last 3 bp of each read were not taken into consideration. In addition, both criteria include that at least a PHRED base quality value of 20, corresponding to an error rate of *≤* 1% must be estimated for the genotype call at that site in that individual.

To select the most proper set of SNPs for the imputation, several cut‐offs were applied based on the minimum call rate (MCR). As a result, imputation was used on the MCR50 SNPs with 32.25% missing data and 1.11% polymorphisms to fill in the gaps where there were insufficient reads to make genotype calls within a genotype. The maximum missing data rate per SNP site was 50% in the MCR50 SNPs (Table 1). Imputation was performed by using BeagleV5.1 (http://faculty.washington.edu/browning/beagle/beagle.html) with 50 phasing iterations and other default parameters. The following criteria in filtering MCR50 SNPs were applied: Minimum calling rate *≥* 50%, allele number = 2, number of genotypes *≥* 2, minor allele frequency (MAF) *≥* 5%, and heterozygosity rate range: 0% – (2 *× Frequency*_*allele*1_ *× Frequency*_*allele*2_ + 20%). To avoid the low accuracy associated with the imputation of scaffold SNPs, the imputation was performed only on the chromosome‐based SNPs.

**TABLE 1:** Summary of SNPs genotyped in different MCR (minimum call rate) levels.

	No. SNPs	Missing data points	% Polymorphisms (raw reads)
MCR50	247,008	21,986,571/68,174,208 = 32.25%	247,008/22,154,507 = 1.11%
MCR60	169,294	12,300,433/46,725,144 = 26.33%	169,294/14,595,270 = 1.16%
MCR70	100,121	5,601,229/27,633,396 = 20.27%	100,121/8,493,851 = 1.18%
MCR80	43,928	1,702,588/12,124,128 = 14.04%	43,928/3,835,705 = 1.15%
MCR90	8,533	166,432/2,355,108 = 7.07%	8,533/886,584 = .96%

### 2.5 SNP analysis

Principal component analysis (PCA) was performed using TASSEL v5 (Bradbury et al., 2007). The eigenvalues of 10 PCs were used to create the scree plot to visualize the proportion of variance explained by each PC. The software PLINK 1.9 (Chang et al., 2015) was utilized in R (R Core Team, 2020) in order to estimate the linkage disequilibrium (LD) with the *r* ^2^ statistics, build LD decay, and perform the minor allele frequency. In addition, the R package LDheatmap was used to plot the heat maps of pairwise LD between SNPs (Shin et al., 2006). Kinship was estimated using TASSEL v5 (Bradbury et al., 2007) and illustrated in Python (Van Rossum & Drake, 1995). In addition, a neighbor‐joining phylogenetic tree was constructed using the distance between each accession as per SNPs with MAF > .05 using the TASSEL plugin “‐tree Neighbor” (TASSEL v5) (Bradbury et al., 2007). The phylogenetic tree was visualized using Interactive Tree of Life (iTOL) (http://itol.embl.de/index.shtml), an online tool for displaying phylogenetic tree (Letunic & Bork, 2021).

### 2.6 Statistical methods for genome‐wide association study

As a first step, 247,008 SNPs were detected and filtered into 246,671 SNPs by removing the excessive alignments on the unplaced genomic scaffolds. For the marker‐trait association analysis, the initial SNP set (246,671 SNPs) was filtered by removing the SNPs with minor allele frequencies of ≤ .05 across the 274 individuals, resulting in a set of 226,779 SNPs. The resulting marker set was employed for GWAS analysis by a single‐locus model; MLM (Q + K) (Price et al., 2006), and a multi‐locus model; farmCPU (Liu et al., 2016). Both models were run using the R package “rMVP” (v1..6) (Yin et al., 2021). For the MLM model, the kinship matrices having the relationship among individuals (VanRaden, 2008) and the first three principal components (PCs) were fit as covariates to control for the confounding effects of the population structure. Following a previous study by Palali Delen et al. (2023). The threshold for the significant association of SNPs was set to 2.2 × 10–7(.05/n, n = 226,779). For the farmCPU model, the kinship matrix calculated internally by the farmCPU algorithm was fitted as random effects in addition to the first three PCs as covariates. Manhattan plots and Q‐Q plots representing GWAS results were plotted in rMVP itself.

## 3 RESULTS

### 3.1 Phenotypic distribution, heritability, and BLUP value calculation

**FIGURE 1:**
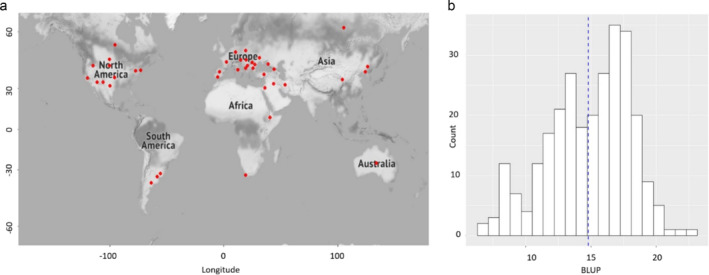
Geographic locations of genotyped sunflower accessions and the distribution of BLUP value. (a) A map illustrating the geographic coordinates of the sunflower accessions. Red dot denotes the location of each accession. (b) The histogram distribution of BLUP values. The blue dashed line shows the mean BLUP value of the disc diameter.

In this study, a set of 342 geographically widely distributed sunflower accessions was obtained from the North Central Regional Introduction Station (NCRIS) (see Table S1). Based on the location information extracted from the Germplasm Resources Information Network (GRIN), the majority (314/342, or 92%) of the selected sunflower accessions originated from North America, Europe, and Asia, and a small subset (28/342, 8%) of accessions are from South America, Africa, Australia, or unknown locations (*n* = 2) (Figure 1a). These accessions were planted in 2019, 2020, and 2022 following an incomplete block design (see Section 2, Figure S1). From the field experiment, the disc diameter was manually measured from up to three representative plants per plot (Figure S2).

In 2019, as a result of more significant damage from sunflower moths, the distribution of observed sunflower disc diameter was skewed towards substantially smaller discs (mean size *≈* 8 cm) as compared to 2020 (mean size *≈* 19 cm) and 2022 (mean size *≈*17 cm) (Figure S3). After analyzing these three years of data, the heritability (*H* ^2^) of disc diameter was estimated as .88. Excluding 2019 data obtained a slightly higher heritability of *H* ^2^ = .92. Because the sunflower moth damage did not dramatically affect the heritability of the trait, we then combined three years of data to calculate BLUP values (Section 2). As a result, BLUP values of the disc diameter trait ranged from 6.8 to 22.8 with a mean of 14.8 (Figure 1b).

### 3.2 SNP genotyping and population structure analysis

For a subset (285/342, or 83%) of the sunflower accessions, we conducted genotyping using the tGBS method (Ott et al., 2017) (see Section 2 and Table S2). After SNP calling, we filtered out SNPs with a minimum calling rate less than 50% and also filtered out 11/285 accessions because of the high individual missing rate (i.e., > 90%). As a result, a set of 247,008 SNPs were retained for 274 sunflower accessions. For this SNP set, the average missing rate is 32.3%, and the average number of reads per SNP site per sample is 22 (Figure S4). After filtering the SNP set into 246,671 SNPs by removing the excessive alignments on the 147 unplaced genomic scaffolds, the minor allele frequencies of ≤ .05 were applied, resulting in a set of 226,779 SNPs. These SNPs were evenly distributed across the 17 sunflower chromosomes (Figure S5).

**FIGURE 2:**
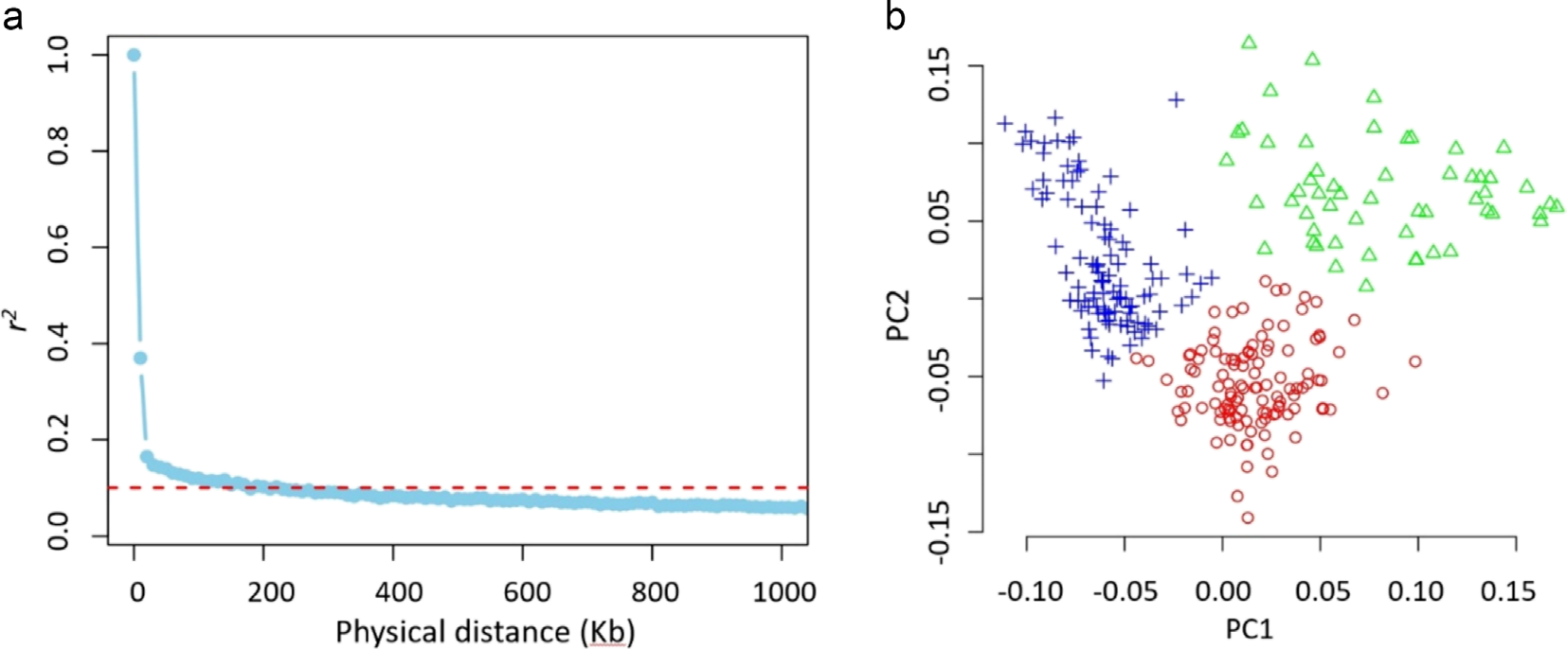
Properties of genotype data. (a) LD decay. The threshold was set to .1. (b) PCA plot. The accessions were clustered by using the simple k‐means algorithm with the three groups.

With the filtered SNP set, we found the LD decays rapidly in the population, i.e., average pairwise SNP distance elevated from 30 kb to 18 kb while the LD reduced from *r* ^2^ = .2 to .15 (Figure 2a). When the LD is *r* ^2^ = .1, the average physical distance between two SNPs is about 220 kb, largely consistent with a previous study (Filippi et al., 2020). Principal component analysis suggested that the first principal component (PC) explained about 5% of the variance and the top 10 PCs explained 25% of the variance in total (Figure S6). Using the k‐means algorithm, three groups were detected (Figure 2b), suggesting our sunflower accessions are likely composed of three sub‐populations. A neighbor‐joining phylogenetic tree was also constructed and visualized using the distance between each accession as per SNPs with MAF > .05 (Figure S7).

### 3.3 GWAS results for the disc diameter trait

**FIGURE 3:**
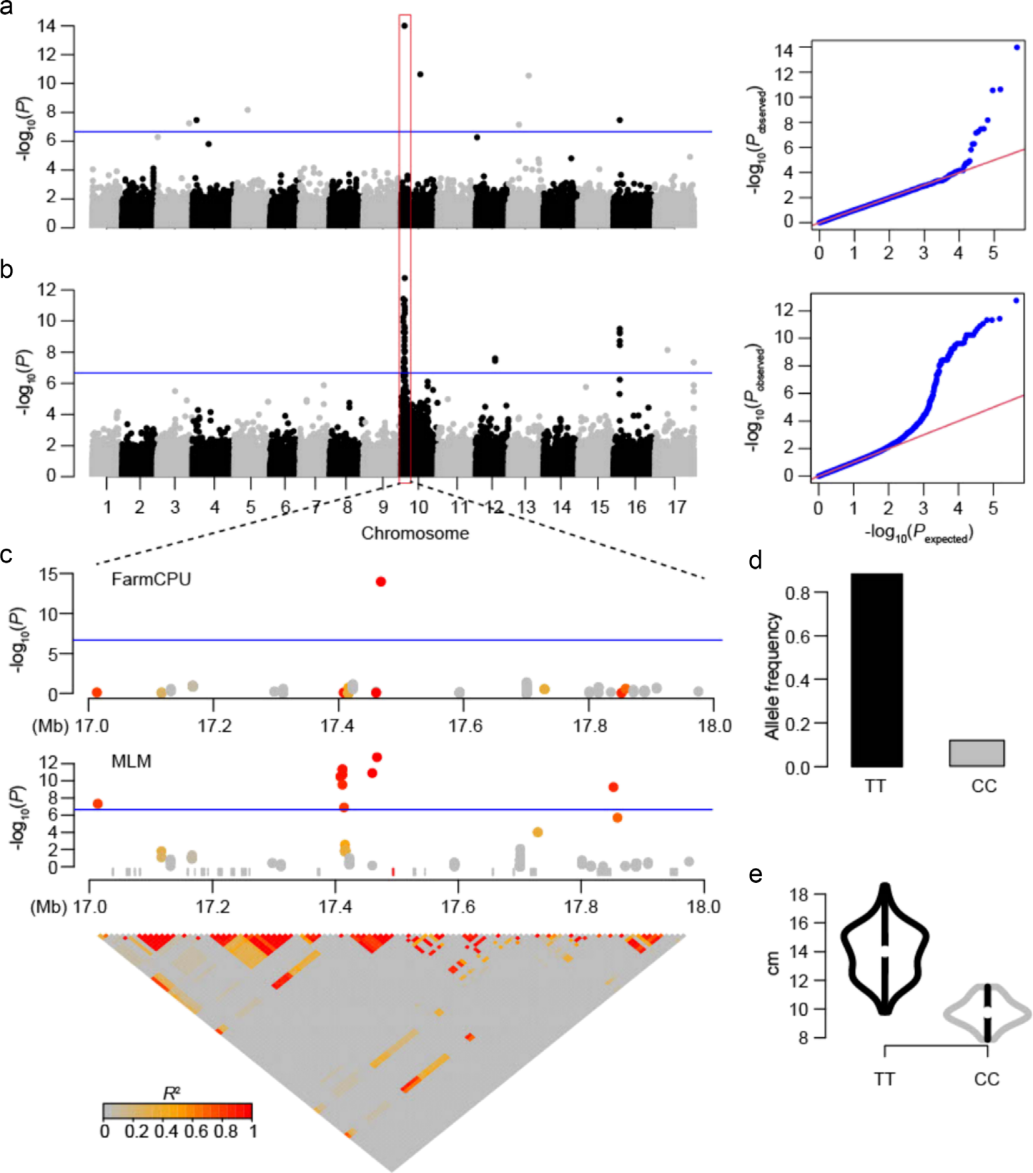
Disc diameter associated genetic loci and the zoom‐in plots on a Chr10 signal. Manhattan plots and quantile‐quantile (Q‐Q) plots by using (a) FarmCPU and (b) MLM methods. The vertical and horizontal axes in the Manhattan plots indicate the P values in the *−log10* scale and the chromosome numbers, respectively. The horizontal blue lines indicate the significance thresholds. (c) A zoom‐in plot of the highlighted (500 kb upstream and downstream of the most important SNP) region on chromosome 10 and LD plot in the highlighted region. In the zoom‐in plot, the gray rectangles represent the annotated gene models. The red one is the gene that is closest (25 kb) to the most significant SNP (NC_035442.2–17,467,721) at chromosome 10. The LD level (r2) of windows with the leading signal is represented by color in the zoom‐in figure. (d) Allele frequency of the most significant SNP detected both in MLM and farmCPU methods. (e) Distribution of the disc diameter trait of sunflower accessions carrying “TT” and “CC” genotypes.

Using the set of 226,779 SNPs, we conducted GWAS with two different statistical methods, i.e., MLM and FarmCPU (see Section 2). Quantile‐quantile (Q‐Q) plots suggested the population structure was well controlled for our GWAS analyses (Figure 3). By setting a stringent Bonferroni adjusted P‐value cutoff (*P* = 2.2 *×* 10^*−*7^ determined by .05/n, n = 226,779), the FarmCPU method identified *n* = 8 significant trait‐associated SNPs located on 6 different chromosomes (Figure 3a, Table S3). These FarmCPU‐identified GWAS SNPs were located close to 21 genes (Table S4). For the MLM method, 106 SNPs were identified that were located close to 53 genes (Table S5, Table S6). n = 5 GWAS peaks were above the Bonferroni threshold (Figure 3b). If requiring at least three significant SNPs within a peak, two significant GWAS peaks were identified, one located on chr10 and the other on chr16. Interestingly, the most significant SNP (NC_035442.2–17,467,721) on chromosome 10 and another significant SNP (NC_035448.2–31,775,666) on chromosome 16 found by the MLM method were also detected by the FarmCPU method.

For the chr10 peak, 86 significant trait‐associated SNPs were identified, ranging across a .9 Mb genomic region from 17.0 Mb to 17.9 Mb (Figure 3c). According to the genome annotation information, 41 genes are located within this genomic region. A candidate gene (Gene ID: 118483017) was located 25 kb away from the most significant SNP (NC_035442.2–17,467,721). The advantage genotype at the most significant GWAS signal is a “TT” genotype, with a frequency of .88 (Figure 3d). The average disc diameter of the sunflower plants carrying the “TT” genotype is 13.93 cm, compared to the average value of 9.78 cm for plants with the “CC” genotype (Figure 3e).

Similarly, for the peak detected on chromosome 16, 10 significant trait‐associated SNPs were identified by using the MLM method, one of which was detected by the FarmCPU method (Figure 3). The MLM GWAS peak ranges across a 2 Mb genomic region from 31.0 to 33.0 Mb on chromosome 16, and 7 genes are located within this genomic region (Figure S8). The “TT” genotype at the most significant GWAS signal (NC_035448.2–31,753,915) has an average disc diameter of 13.86 cm with a frequency of .91 as compared to the “AA” genotype with an average value of 9.95 cm with a frequency of .09.

## 4 DISCUSSION

Sunflower disc is an important trait in sunflower populations, directly impacting seed yield. As the sunflower disc size increases, so do the number of seeds and the 100‐seed weight. Multiple studies have reported a positive correlation between sunflower disc diameter, the number of seeds, and 100‐seed weight, underscoring their critical roles in influencing seed yield (Clapco et al., 2019; Machikowa & Saetang, 2008; Radić et al., 2021; Yasin & Singh, 2010). The previous study suggested that sunflower disc diameter is a quantitative trait governed by complex genetic mechanisms controlled by numerous genes and their interactions with the environments (Sham et al., 2002). Therefore, investigating the genetic basis of sunflower disc diameter requires a multidisciplinary approach.

Recent technological advancements have facilitated wide genetic research on yield‐related traits in economically important crops like wheat, rice, and maize (Ashfaq et al., 2023; Dwiningsih & Al‐Kahtani, 2022; Khan et al., 2022; Lv et al., 2022; Ndlovu et al., 2022; Rathan et al., 2022; Soleimani et al., 2022; Wang et al., 2023; Zeng et al., 2022). It is now simpler to acquire faster and lower‐cost sequencing tools with higher throughput data (Ashraf et al., 2022; Bielecka et al., 2022; de la Fuente Cantó & Vigouroux, 2022; Park & Kim, 2016), leading to the latest valuable studies on sunflower (Akter et al., 2024; Ding et al., 2024; Sami et al., 2024; Song et al., 2024). Despite that, genome‐wide association studies on sunflower disc diameter have been limited, leaving a gap in existing knowledge. In this study, we evaluated a set of diverse sunflower accessions that were selected from different regions of the world for the disc diameter trait in replicated field trials over three years. In our population, we discovered significant phenotypic variation in disc diameter, as was reported by Onemli and Gucer (2010) (Onemli & Gucer, 2010), Balalic´ et al. (2016), and Dowell et al. (2019). The disc diameter values in the population ranged from 3.00 cm to 20.00 cm with a mean of 8.05 cm in 2019, from 7.25 cm to 29.80 cm with a mean of 19.03 cm in 2020, and from 5.50 cm to 30.00 cm with a mean of 17.29 cm in 2022. In addition, the trait was highly heritable with an estimated heritability of .88, consistent with previous studies (Jocković et al., 2013). For these diverse accessions, we generated a high‐density SNP panel composed of more than 200 k high‐quality SNPs. As compared to other crop species, the genetic and genomic resources are limited in sunflowers. In addition to some of those genotyped sunflower populations reported in other studies (Badouin et al., 2017; Chernova et al., 2021; Mandel et al., 2011; Todesco et al., 2020), genomic data of our population that we presented in this study will be a good source that can be extensively used by the sunflower research community. These diverse sunflower accessions and the high‐quality SNP set provide invaluable resources for population genetic analysis in sunflowers.

Through a combined analysis of the SNPs and the disc diameter phenotype, two trait‐associated loci were repeatedly detected using two different GWAS methods. The Chr10 locus can elevate the disc diameter by 4 cm, and the Chr16 locus increases by 3 cm. In addition to these two strong GWAS signals, a number of less significant GWAS peaks were identified. Based on these results, markers can be developed to facilitate marker‐assisted selection. It is anticipated that only by employing the two markers targeting the two major effect loci, a maximum improvement of 7 cm of disc diameter can be achieved for a breeding program in which these two loci are dominant by the unfavorable alleles.

In this study, in addition to the trait‐associated SNPs, we identified several genes underlying the GWAS peaks with a total of 21 and 53 genes by using farmCPU and MLM, respectively. Two and forty of those genes were closely located to the significant SNPs detected by farmCPU and MLM methods on chromosome 10. Similarly, in another study conducted by Wills and Burke (2007) (Wills & Burke, 2007), QTLs that are associated with the disk diameter and other floral traits (number of heads and seed shattering) on chromosome 10 were reported. Those QTLs were closely located to the ORS437 marker, placed on the 17,1 cM of chromosome 10. In the following years, Wills et al. (2010) (Wills et al., 2010) identified QTLs for various achene traits, including achene size and weight, nearly located to the marker 0RS437. Since there is a high correlation between the disc size and the number of seeds (Alkio et al., 2003; Idrees et al., 2023; Marinković, 1992; Palmer & Steer, 1985), the genes controlling head diameter may also be influencing the seed characters.

Many of the genes we identified have no clear functional assignment due to the limited functional study in sunflowers. To delineate the relationship between the trait‐associated loci and the disc diameter trait variation, further molecular characterization is warranted. Some other genes that were closely located to the SNPs detected by MLM and farmCPU methods were functional for coding proteins (Table S7). Among those, some of the proteins coded by the identified genes stand out for their roles in plant development, impacting floral organ size and yield. For instance, the transcription repressor MYB6 protein that is coded by the LOC110930217 gene, which we detected closely located to the SNP “NC_035435.2‐160,345,299”, was found to be related to the flavonoid biosynthetic gene expression, leading to a noticeably higher anthocyanin and proanthocyanidin accumulation (Wang et al., 2019). It is known that flavonoids are powerful regulators of auxin (Besseau et al., 2007; Buer et al., 2013; Doughty et al., 2014; Jacobs & Rubery, 1988; Peer et al., 2001), which plays a variety of functions in the growth and development of plants, including meristem maintenance, cell patterning, organogenesis, and cell division (Perrot‐Rechenmann, 2010). Another protein coded by the gene “LOC110903133” we detected closely located to the SNP “NC_035436.2‐20,355,884” was an NLP7‐like protein. According to several studies (Guan et al., 2017; Zhao et al., 2018), NLP7 is a key regulator of nitrate signaling. In addition, a substantial interaction between auxin transport and nitrate assimilation and signaling was reported (Asim et al., 2020; Liu & Von Wirén, 2022). This is important because auxins support plant development, especially during the flowering time. According to Clifford et al. (1992), the process is aided by an increase in plant hormone levels, such as auxin, during the time of inflorescence formation. A study by Pandey et al. (2003) found that when cotton plants were treated with synthetic auxin, a notable rise was observed in the quantity and weight of flowers. Likewise, Jamil et al. (2016) reported the positive effect of synthetic IAA auxin in the number and size of flowers of Hippeastrum (*Hippeastrum hybridum* Hort.). 1‐aminocyclopropane‐1‐carboxylate oxidase homolog 1 was also another protein coded by the gene “LOC110884013” that we detected related to sunflower disc diameter. Some studies reported that this protein played a significant role during the flowering process. Barry et al. (1996) (Barry et al., 1996) indicated that the genes coding those proteins were expressed during the flowering stage of the tomato plant. Ruduś et al. (2013) also supported this by indicating the contribution of the 1‐aminocyclopropane‐1‐carboxylate oxidase gene to the last stage of ethylene synthesis in the tissues of plants, playing significant roles during flower development (Martínez et al., 2022). The GRF‐interacting factor (GIF) 1 protein was found to act in the development of plant organs, including flowers. Findings by Lee et al. (2009) revealed that the expression patterns of *GIF* genes overlap in numerous tissues and that *gif* mutations work in concert to cause a significant decrease in the number of cells in lateral organs like leaves, flowers, and cotyledons, causing smaller plants in Arabidopsis. A similar result was also reported previously by Zhang et al. (2018) in maize. The F‐box protein family members have a role in the control of numerous important physiological functions, including plant growth and development and the reaction to environmental cues (Xu et al., 2021). In this study, we revealed that the gene “LOC110880639” was functional for coding putative F‐box only protein 15. It was reported that some F‐box genes are responsible for affecting the flower size in various plant species (Damayanti et al., 2019; González‐Carranza et al., 2007; González‐Carranza et al., 2017; Levin & Meyerowitz, 1995; Zhao et al., 2001). We also found that some genes were responsible for coding zinc finger proteins (zinc finger CCCH domain‐containing protein 6 and zinc finger protein 11‐like) that play essential roles in plant development (Feurtado et al., 2011). It was found that zinc finger proteins regulate the floral organ size (Disch et al., 2006) and flowering time (Chen & Ni, 2006) in Arabidopsis. Another protein that plays an important role in plant development (Haffani et al., 2006; Shumayla et al., 2022) was proline‐rich protein 36‐like coded by the gene “LOC110919168,” which we detected related to the disc diameter of sunflower.

Sunflower demand has been rising due to the growing human population and other environmental concerns. To meet this demand, sunflower seed yield has to be increased over time. In the scope of this study, the significant SNPs and the candidate genes we detected are of significant importance in their function in coding proteins that influence the sunflower disc diameter, directly affecting sunflower seed yield. Due to the limited number of genome‐wide association studies on the disc diameter trait of sunflowers, our findings in this study are essential. The identified GWAS signals and candidate genes can be leveraged to improve the yield and quality of sunflowers and address increasing needs.

## AUTHOR CONTRIBUTIONS

**Yavuz Delen**: Data curation, investigation, methodology, visualization, formal analysis, writing—original draft preparation. **Ravi V. Mural**: Formal analysis, visualization, validation, writing—review and editing. **Gen Xu**: Formal analysis, visualization, writing—review and editing. **Semra Palali‐Delen**: Investigation, methodology, formal analysis, writing—review and editing. **James C. Schnable**: Writing—review and editing. **Ismail Dweikat**: Project administration, supervision, conceptualization, resources, writing—review and editing. **Jinliang Yang**: Supervision, investigation, validation, writing—review and editing. All authors reviewed the manuscript.

## CONFLICT OF INTEREST STATEMENT

The Authors did not report any conflict of interest.

## Supporting information


**Data S1.** Peer review.

**Figure S1.** Overview of the field experimental design. An incomplete block design was used with two main blocks (only one block in 2019), four split plots per block, and three replicates of the check (PI 432513) per split‐plot.

**Figure S2.** Planting design and phenotype data collection procedure. Head diameter (in centimeters) was measured for three representative plants per plot, excluding edge plants.

**Figure S3.** The distribution of average head sizes in 2019 (A) (Average of 3 measurements), 2020 (B) (Average of 6 measurements −2 plots * 3 measurements‐), and 2022 (Average of 6 measurements −2 plots * 3 measurements‐). The vertical and horizontal axes indicate the number of accessions and the head diameter measurements in cm, respectively. The blue dashed lines show the mean values.

**Figure S4.** Summary of the genotyping results. (A) Distribution of number of reads per sample. (B) Distribution of the number of reads per interrogated base per sample. (C) Distribution of average missing Rate per SNP site. (D) Distribution of the number of reads per SNP site per genotyped sample.

**Figure S5.** The SNP density distribution on genotyped sunflower population. The number of SNPs within 1 Mb window size through sunflower chromosomes.

**Figure S6.** Distribution of explained variance of PCA by component. SNP variation explained by first 10 PCs.

**Figure S7.** Phylogenetic tree. The phylogenetic tree was constructed using the distance between each accession as per SNPs with MAF > .05.

**Figure S8.** The zoom‐in plots of the highlighted (500 kb upstream and downstream of the shared SNP “NC_035448.2–31,775,666” by farmCPU (A) and MLM (B) methods) region on chromosome 16. The vertical and horizontal axes indicate the P values in −log10 scale and the chromosomal positions, respectively. The points are the SNPs and the blue horizontal line indicates the genome‐wide thresholds of Bonferroni correction (P < 2.2e − 7). The gray rectangles represent the gene models that are annotated. The red one is the gene “LOC110919168”, which is closest to the shared SNP at chromosome 16.

**Table S1.** 342 Accessions Phenotyped. A total of 342 accessions were evaluated in 2019, 2020, and 2022 that were provided by the United States Department of Agriculture, Agricultural Research Center (USDA‐ARS), North Central Regional Introduction Station (NCRPIS) in Ames, Iowa, USA.

**Table S2.** Genotyped accessions by tGBS. A total of 274 sunflower accessions collected in different regions of the world and stored at the USDA‐ARS, NCRPIS were genotyped by tGBS technology.

**Table S3.** Significant SNPs determined by farmCPU. The table shows the significant SNPs with position (POS), reference (REF), and alternative (ALT) alleles, SNP effect, standard error (SE), and p‐value detected by farmCPU method.

**Table S4.** Gene annotation with significant SNPs detected by farmCPU. In addition to the detected significant SNPs by farmCPU, it includes the information of genes annotated. Gene annotation was performed using the previously detected genes (http://ftp.ensemblgenomes.org/pub/plants/release-52/gff3/helianthus_annuus/) after applying 50 kb of upstream and downstream to the detected significant SNP positions.

**Table S5.** Significant SNPs determined by MLM. The table shows the significant SNPs with position (POS), reference (REF), and alternative (ALT) alleles, SNP effect, standard error (SE), and p‐value detected by the MLM method.

**Table S6.** Gene annotation with significant SNPs detected by MLM. In addition to the detected significant SNPs by MLM, it includes the information of genes annotated. Gene annotation was performed using the previously detected genes (http://ftp.ensemblgenomes.org/pub/plants/release-52/gff3/helianthus_annuus/) after applying 50 kb of upstream and downstream to the detected significant SNP positions.

**Table S7.** Genes functional for coding proteins. The table shows the genes closely located to the SNPs detected by farmCPU and MLM methods.

## Data Availability

The data for a set of 247,008 SNPs retained after filtering out with a minimum calling rate of more than 50% (MCR50) and 226,779 SNPs that were used for GWAS after applying the MAF of ≤ .05 are available at https://github.com/ydelen2/Sunflower_important_traits/tree/main/Genotype_data.
