# Supplementary material for: Dissecting the genetic architecture of sunflower disc diameter using genome‐wide association study
Source: Plant Direct. 2024 Oct 9;8(10):e70010. doi: 10.1002/pld3.70010 (PMC11464090; doi:10.1002/pld3.70010)
Supplement: Supplementary file 2 — Figure S1. Overview of the field experimental design. An incomplete block design was used with two main blocks (only one block in 2019), four split plots per block, and three replicates of the check (PI 432513) per split‐plot. [file PLD3-8-e70010-s013.docx]

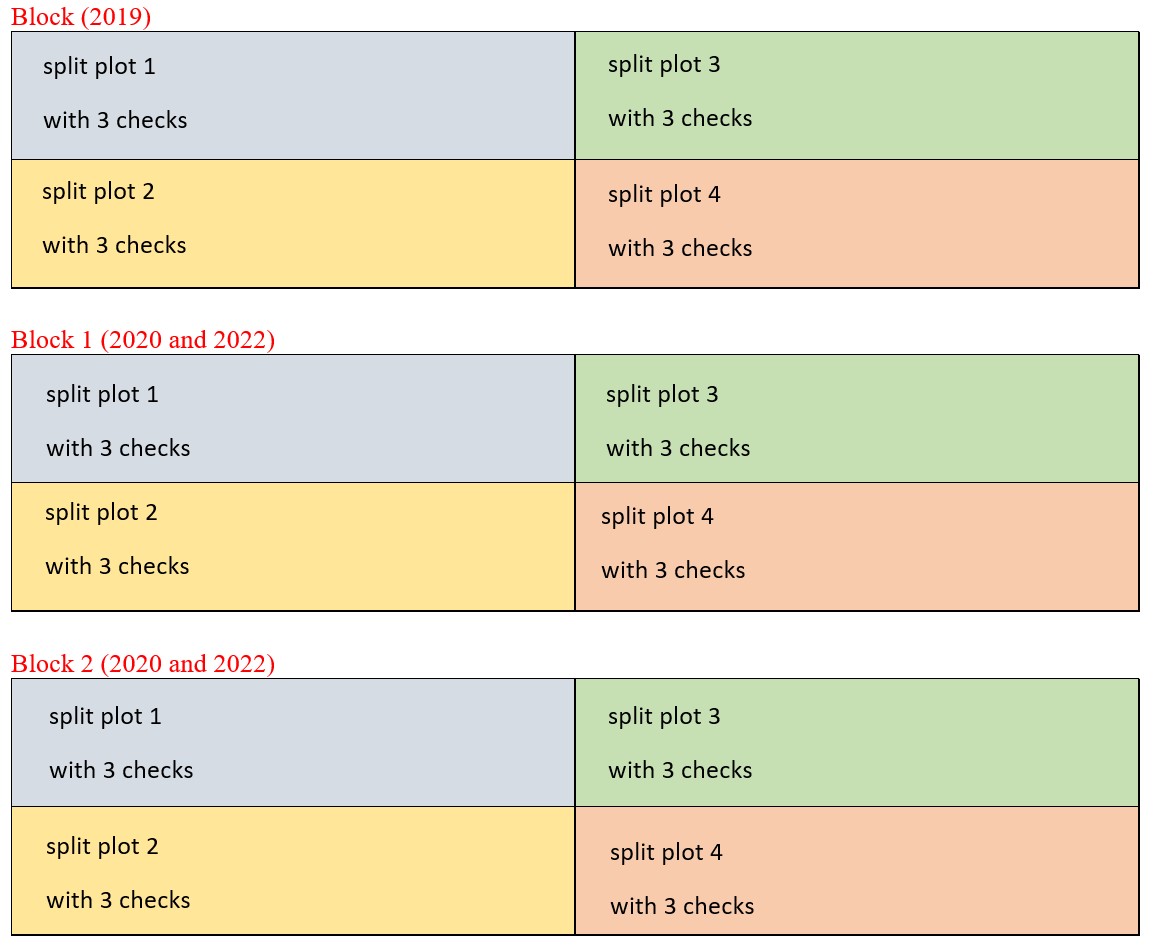


**Figure S1. Overview of the field experimental design.** An incomplete block design was used with two main blocks (only one block in 2019), four split plots per block, and three replicates of the check (PI 432513) per split plot.
