# Supplementary material for: Dissecting the genetic architecture of sunflower disc diameter using genome‐wide association study
Source: Plant Direct. 2024 Oct 9;8(10):e70010. doi: 10.1002/pld3.70010 (PMC11464090; doi:10.1002/pld3.70010)
Supplement: Supplementary file 3 — Figure S2. Planting design and phenotype data collection procedure. Head diameter (in centimeters) was measured for three representative plants per plot, excluding edge plants. [file PLD3-8-e70010-s004.docx]

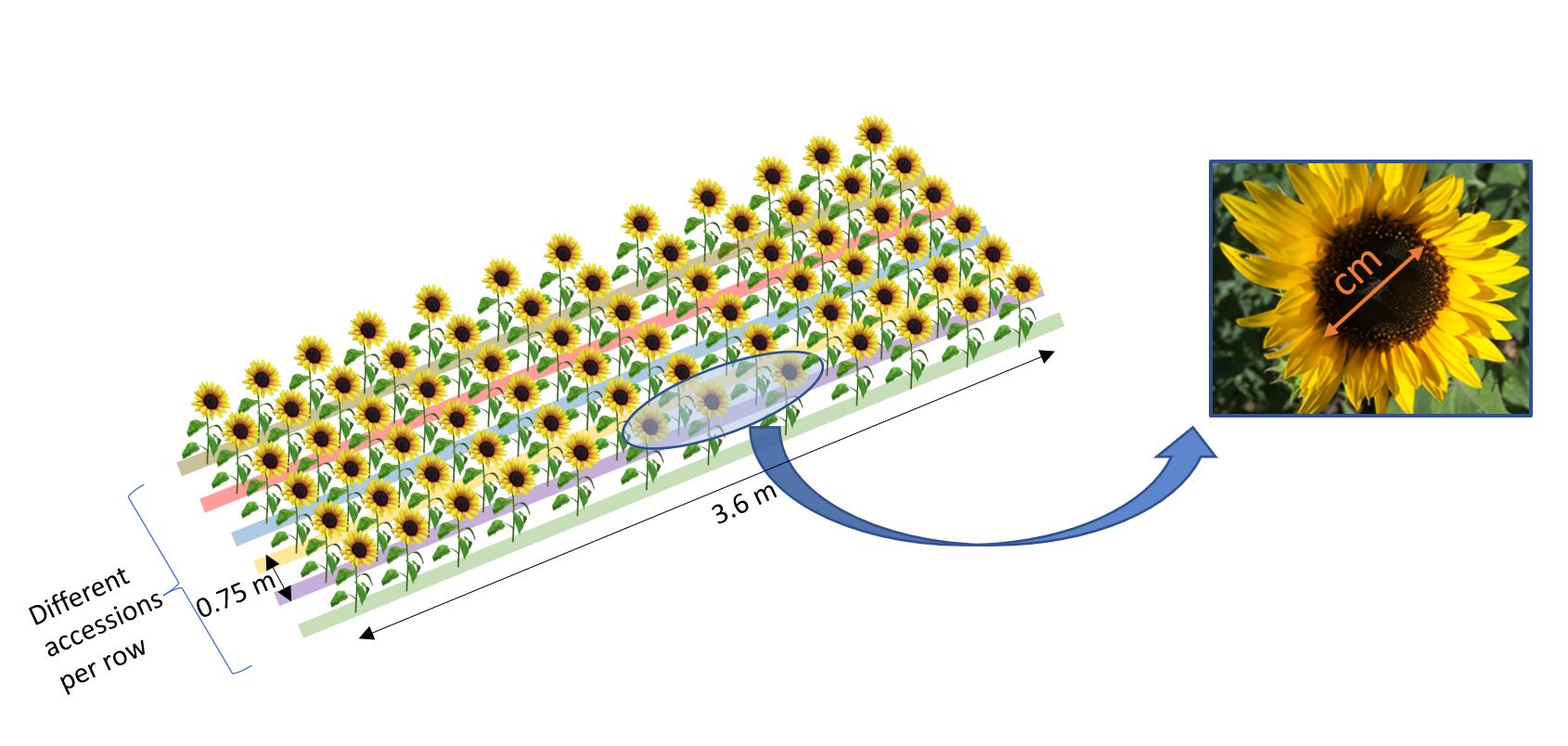


**Figure S2. Planting design and phenotype data collection procedure.** Head diameter (in centimeters) was measured for three representative plants per plot, excluding edge plants.
