# Supplementary material for: Dissecting the genetic architecture of sunflower disc diameter using genome‐wide association study
Source: Plant Direct. 2024 Oct 9;8(10):e70010. doi: 10.1002/pld3.70010 (PMC11464090; doi:10.1002/pld3.70010)
Supplement: Supplementary file 4 — Figure S3. The distribution of average head sizes in 2019 (A) (Average of 3 measurements), 2020 (B) (Average of 6 measurements −2 plots * 3 measurements‐), and 2022 (Average of 6 measurements −2 plots * 3 measurements‐). The vertical and horizontal axes indicate the number of accessions and the head diameter measurements in cm, respectively. The blue dashed lines show the mean values. [file PLD3-8-e70010-s014.docx]

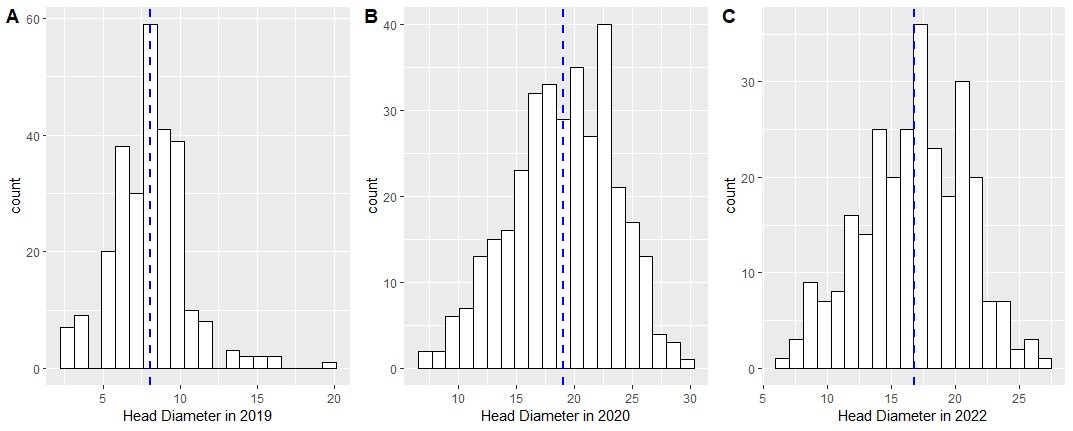


**Figure S3. The distribution of average head sizes in 2019 (A) (Average of 3 measurements), 2020 (B) (Average of 6 measurements -2 plots * 3 measurements-), and 2022 (Average of 6 measurements -2 plots * 3 measurements-)**. The vertical and horizontal axes indicate the number of accessions and the head diameter measurements in cm, respectively. The blue dashed lines show the mean values.
