# Supplementary material for: Dissecting the genetic architecture of sunflower disc diameter using genome‐wide association study
Source: Plant Direct. 2024 Oct 9;8(10):e70010. doi: 10.1002/pld3.70010 (PMC11464090; doi:10.1002/pld3.70010)
Supplement: Supplementary file 5 — Figure S4. Summary of the genotyping results. (A) Distribution of number of reads per sample. (B) Distribution of the number of reads per interrogated base per sample. (C) Distribution of average missing Rate per SNP site. (D) Distribution of the number of reads per SNP site per genotyped sample. [file PLD3-8-e70010-s015.docx]

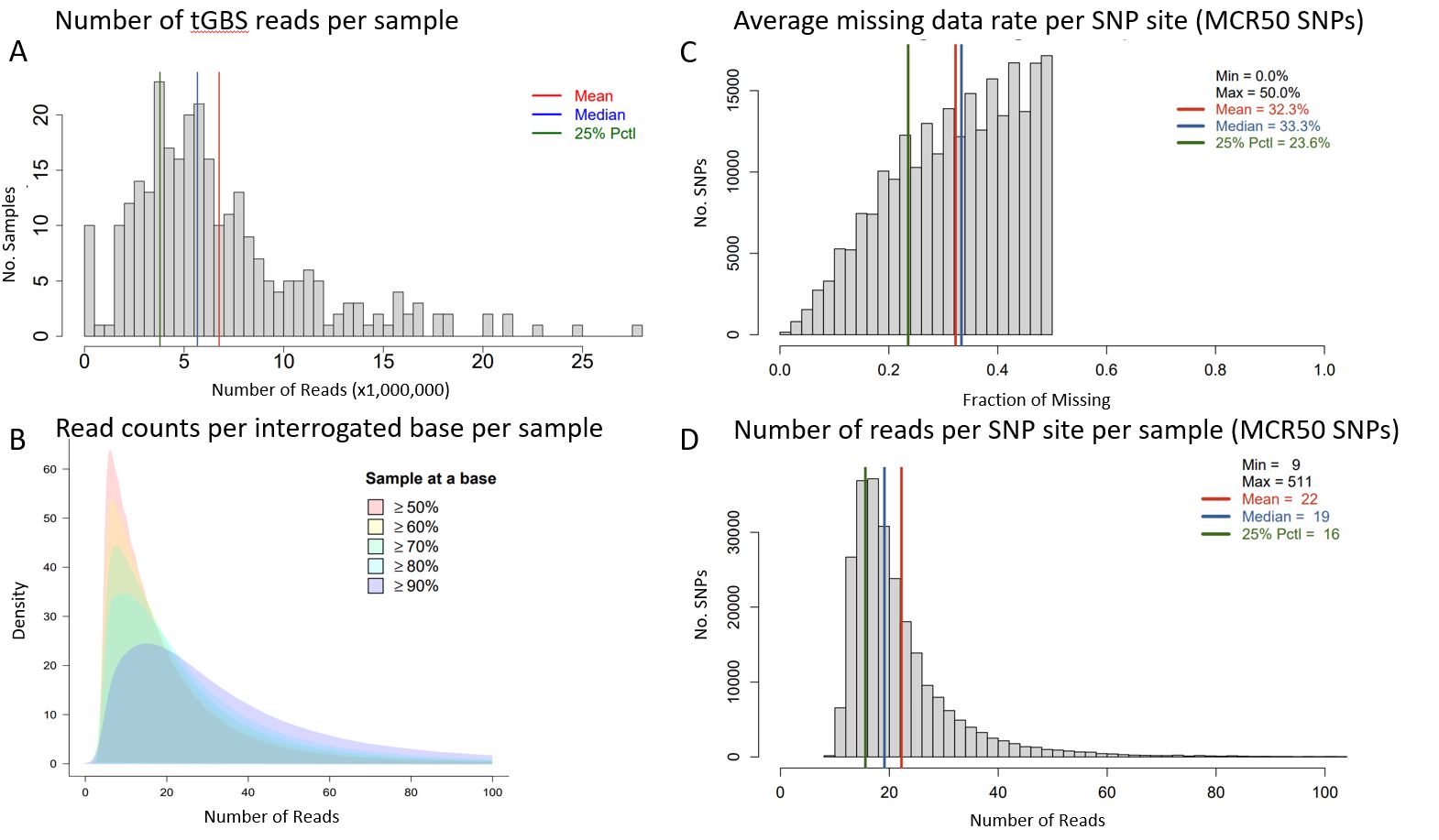


**Figure S4. Summary of the genotyping results. (A)** Distribution of number of reads per sample. **(B)** Distribution of the number of reads per interrogated base per sample. **(C)** Distribution of average missing Rate per SNP site. **(D)** Distribution of the number of reads per SNP site per genotyped sample.
