# Supplementary material for: Dissecting the genetic architecture of sunflower disc diameter using genome‐wide association study
Source: Plant Direct. 2024 Oct 9;8(10):e70010. doi: 10.1002/pld3.70010 (PMC11464090; doi:10.1002/pld3.70010)
Supplement: Supplementary file 6 — Figure S5. The SNP density distribution on genotyped sunflower population. The number of SNPs within 1 Mb window size through sunflower chromosomes. [file PLD3-8-e70010-s006.docx]

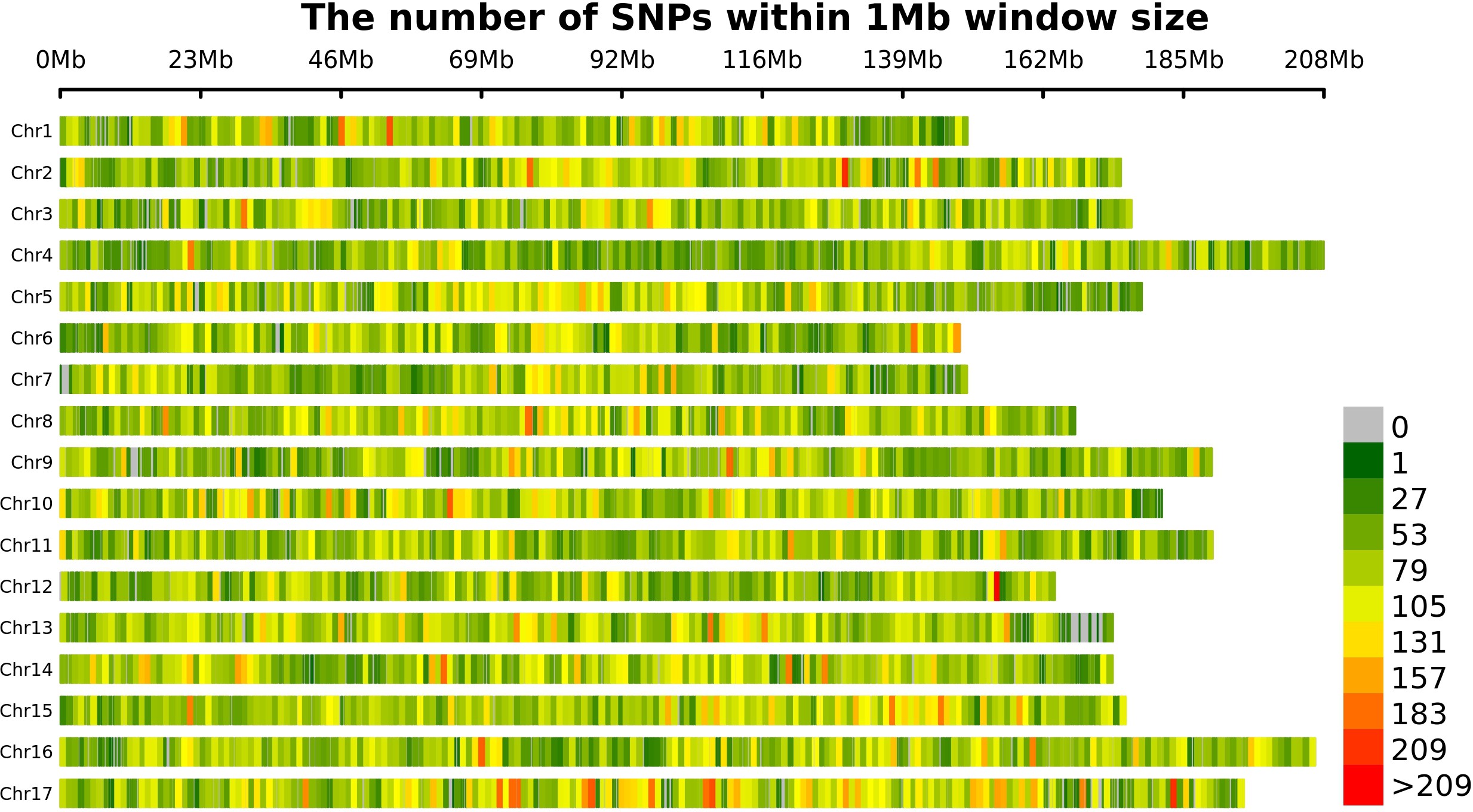


**Figure S5. The SNP density distribution on genotyped sunflower population.** The number of SNPs within 1 Mb window size through sunflower chromosomes.
