# Supplementary material for: Dissecting the genetic architecture of sunflower disc diameter using genome‐wide association study
Source: Plant Direct. 2024 Oct 9;8(10):e70010. doi: 10.1002/pld3.70010 (PMC11464090; doi:10.1002/pld3.70010)
Supplement: Supplementary file 7 — Figure S6. Distribution of explained variance of PCA by component. SNP variation explained by first 10 PCs. [file PLD3-8-e70010-s008.docx]

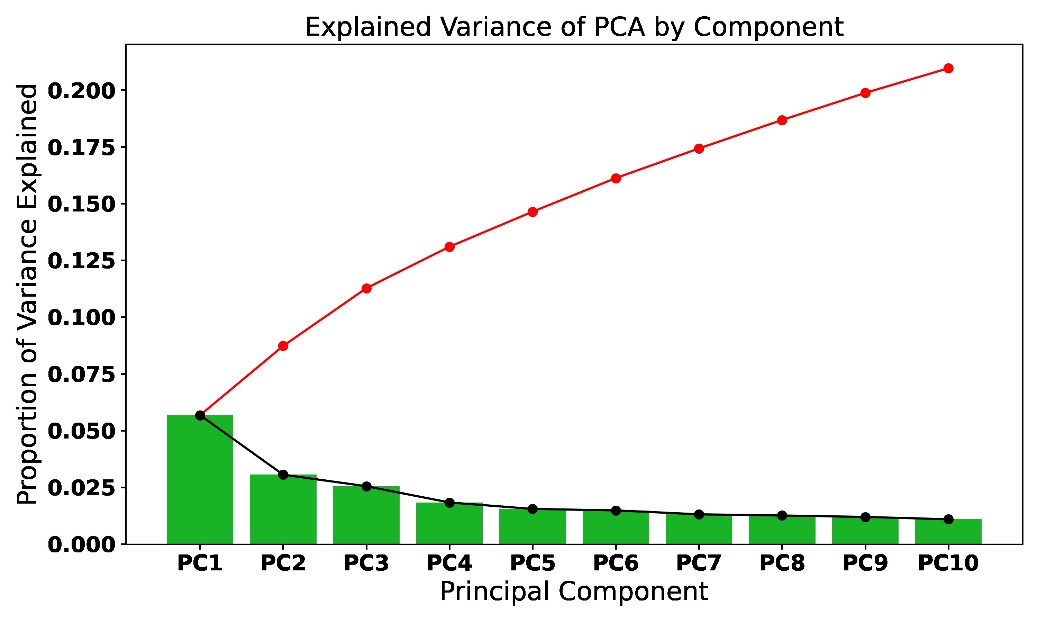


**Figure S6. Distribution of explained variance of PCA by component.** SNP variation explained by first 10 PCs.
