# Supplementary material for: Dissecting the genetic architecture of sunflower disc diameter using genome‐wide association study
Source: Plant Direct. 2024 Oct 9;8(10):e70010. doi: 10.1002/pld3.70010 (PMC11464090; doi:10.1002/pld3.70010)
Supplement: Supplementary file 8 — Figure S7. Phylogenetic tree. The phylogenetic tree was constructed using the distance between each accession as per SNPs with MAF > .05. [file PLD3-8-e70010-s005.docx]

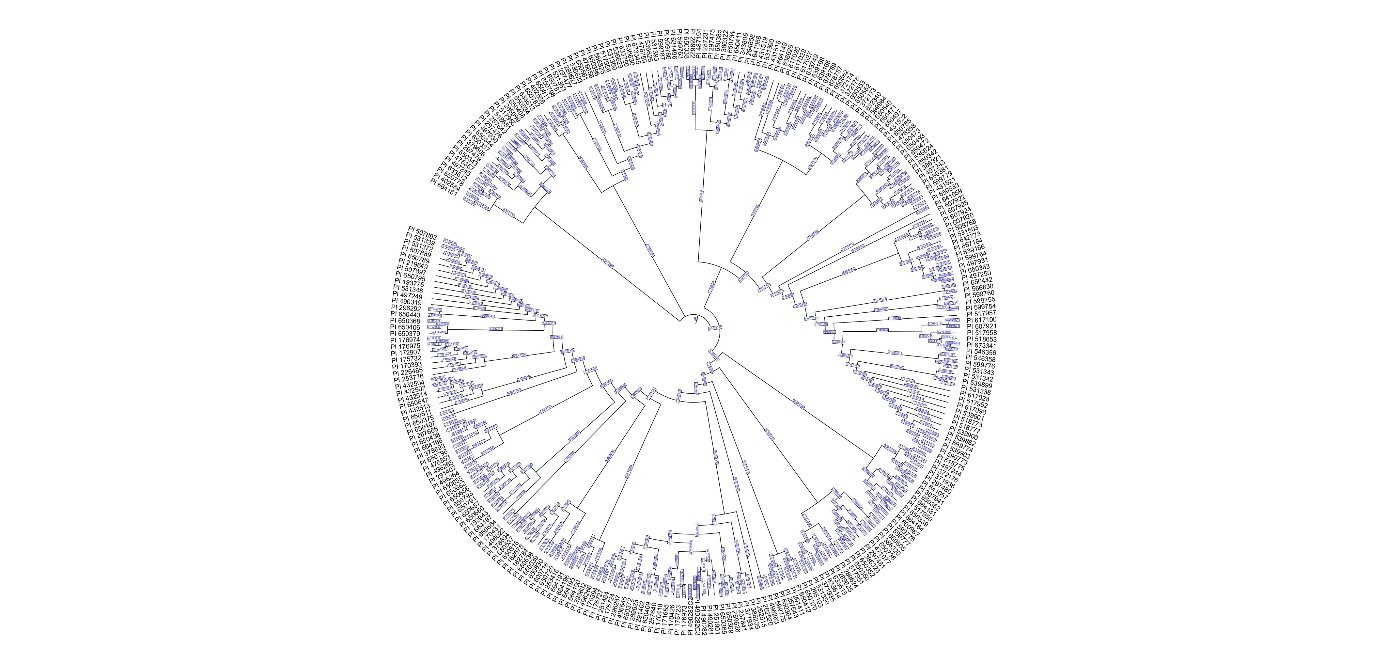


**Figure S7. Phylogenetic tree.** The phylogenetic tree was constructed using the distance between each accession as per SNPs with MAF > 0.05.
