# Supplementary material for: Dissecting the genetic architecture of sunflower disc diameter using genome‐wide association study
Source: Plant Direct. 2024 Oct 9;8(10):e70010. doi: 10.1002/pld3.70010 (PMC11464090; doi:10.1002/pld3.70010)
Supplement: Supplementary file 9 — Figure S8. The zoom‐in plots of the highlighted (500 kb upstream and downstream of the shared SNP “NC_035448.2–31,775,666” by farmCPU (A) and MLM (B) methods) region on chromosome 16. The vertical and horizontal axes indicate the P values in −log10 scale and the chromosomal positions, respectively. The points are the SNPs and the blue horizontal line indicates the genome‐wide thresholds of Bonferroni correction (P < 2.2e − 7). The gray rectangles represent the gene models that are annotated. The red one is the gene “LOC110919168”, which is closest to the shared SNP at chromosome 16. [file PLD3-8-e70010-s009.docx]

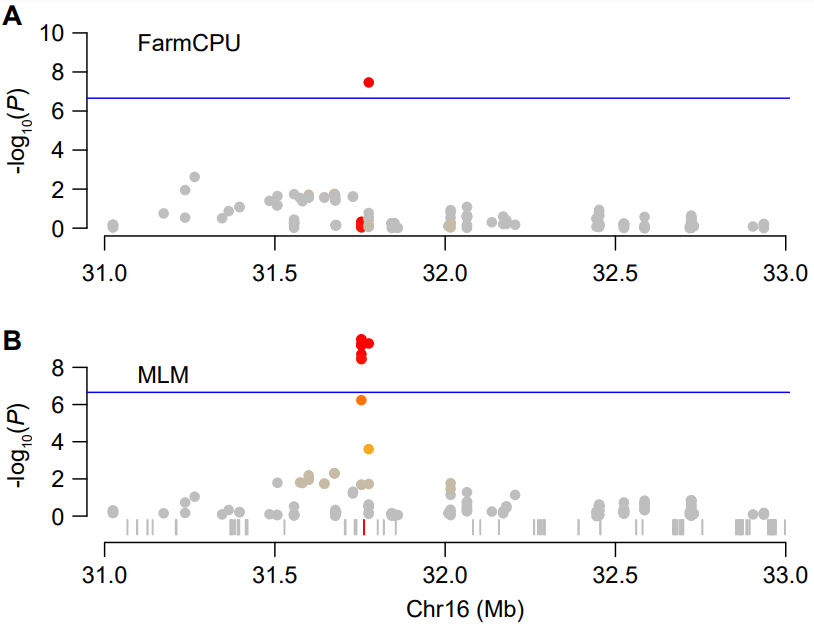


## Figure S8. The zoom-in plots of the highlighted (500 kb upstream and downstream of the shared SNP "NC_035448.2-31775666" by farmCPU (A) and MLM (B) methods) region on chromosome 16. The vertical and horizontal axes indicate the *P* values in *−log*_10_ scale and the chromosomal positions, respectively. The points are the SNPs and the blue horizontal line indicates the genome-wide thresholds of Bonferroni correction (*P <* 2*.*2*e−* 7). The gray rectangles represent the gene models that are annotated. The red one is the gene "LOC110919168" that is closest to the shared SNP at chromosome 16.
