# Supplementary material for: Dissecting the genetic architecture of sunflower disc diameter using genome‐wide association study
Source: Plant Direct. 2024 Oct 9;8(10):e70010. doi: 10.1002/pld3.70010 (PMC11464090; doi:10.1002/pld3.70010)
Supplement: Supplementary file 16 — Table S7. Genes functional for coding proteins. The table shows the genes closely located to the SNPs detected by farmCPU and MLM methods. [file PLD3-8-e70010-s007.docx]

# Table S7. Genes functional for coding proteins. The table shows the genes closely located to the SNPs detected by farmCPU and MLM methods.

| Chr | SNP-Position | GENE | Protein | Method |
| --- | --- | --- | --- | --- |
| 3 | NC_035435.2-160345299 | LOC110930217 | transcription repressor MYB6 | FarmCPU |
| 4 | NC_035436.2-20355884 | LOC110899024 | deoxycytidylate deaminase | FarmCPU |
| 4 | NC_035436.2-20355884 | LOC110903133 | protein NLP7-like | FarmCPU |
| 4 | NC_035436.2-20355884 | LOC110899027 | protein nuclear fusion defective 4 | FarmCPU |
| 4 | NC_035436.2-20355884 | LOC110899025 | TITAN-like protein | FarmCPU |
| 13 | NC_035445.2-52020671 | LOC110897855 | GMP synthase [glutamine-hydrolyzing] | FarmCPU |
| 13 | NC_035445.2-52020671 | LOC110897857 | kinesin-like protein KIN-14J | FarmCPU |
| 13 | NC_035445.2-100405520 | LOC110898814 | uncharacterized protein At4g19900 | FarmCPU |
| 16 | NC_035448.2-31775666 | LOC110919168 | proline-rich protein 36-like | FarmCPU |
| 16 | NC_035448.2-31775666 | LOC110917719 | zinc finger CCCH domain-containing protein 6 | FarmCPU |
| 10 | NC_035442.2-12398748 | LOC110884013 | 1-aminocyclopropane-1-carboxylate oxidase homolog 1 | MLM |
| 10 | NC_035442.2-12398748 | LOC118483367 | 50S ribosomal protein L33-like | MLM |
| 10 | NC_035442.2-10938079 | LOC110880718 | actinidain-like | MLM |
| 10 | NC_035442.2-12398748 | LOC110884011 | autophagy-related protein 9 | MLM |
| 10 | NC_035442.2-12398748 | LOC110884012 | CDT1-like protein a, chloroplastic | MLM |
| 10 | NC_035442.2-15143119 | LOC110880649 | extensin-like | MLM |
| 10 | NC_035442.2-17408177 | LOC110884113 | F-box/kelch-repeat protein SKIP30 | MLM |
| 10 | NC_035442.2-12812961 | LOC110884019 | GRF-interacting factor 1 | MLM |
| 10 | NC_035442.2-19651258 | LOC110884135 | probable aspartic proteinase GIP2 | MLM |
| 10 | NC_035442.2-17013542 | LOC110880648 | protein crowded nuclei 2-like | MLM |
| 10 | NC_035442.2-12398748 | LOC110884010 | protein kinesin light chain-related 1 | MLM |
| 10 | NC_035442.2-10938079 | LOC110883981 | protein NPGR2 | MLM |
| 10 | NC_035442.2-15271146 | LOC110880782 | proton pump-interactor 1-like | MLM |
| 10 | NC_035442.2-15271146 | LOC110880639 | putative F-box only protein 15 | MLM |
| 10 | NC_035442.2-12398748 | LOC110884009 | RING-H2 finger protein ATL1 | MLM |
| 10 | NC_035442.2-14773447 | LOC110880637 | S-protein homolog 2-like | MLM |
| 10 | NC_035442.2-15271146 | LOC118482597 | S-protein homolog 5-like | MLM |
| 10 | NC_035442.2-17013542 | LOC110884070 | sulfate transporter 3.1 | MLM |
| 10 | NC_035442.2-19651258 | LOC110884137 | transcription factor LRL2 | MLM |
| 10 | NC_035442.2-12812961 | LOC110880581 | zinc finger protein 11-like | MLM |
| 16 | NC_035448.2-31753915 | LOC110919168 | proline-rich protein 36-like | MLM |
| 16 | NC_035448.2-31753915 | LOC110917719 | zinc finger CCCH domain-containing protein 6 | MLM |
| 17 | NC_035449.2-193166964 | LOC110886128 | cation/H(+) antiporter 24 | MLM |
| 17 | NC_035449.2-193166964 | LOC110886130 | transcription factor IIIA | MLM |
