# Supplementary material for: Dissecting the genetic architecture of sunflower disc diameter using genome‐wide association study
Source: Plant Direct. 2024 Oct 9;8(10):e70010. doi: 10.1002/pld3.70010 (PMC11464090; doi:10.1002/pld3.70010)
Supplement: Supplementary file 1 — Data S1. Peer review. [file PLD3-8-e70010-s010.pdf]

Plant Direct
Open Access

Dissecting the genetic architecture of sunflower disc diameter using genome-wide association study

Yavuz Delen[1], Ravi V. Mural[2], Semra Palali-Delen[1,3], Gen Xu[1,3], James C. Schnable[1,3], Ismail Dweikat[1], and Jinliang Yang[1,3,*]

Decision Letter Round 1:

May 6, 2024

Jinliang Yang
University of Nebraska-Lincoln
363 Keim Hall
Lincoln, Nebraska 68583

RE: Dissecting the genetic architecture of sunflower disc diameter using genome-wide association study

Dear Dr. Yang:

Thank you for submitting to Plant Direct. All required reviews have been returned and we have now finished our evaluation of your manuscript. In light of the reviewers' and editor's comments, further revisions are needed before the paper can be accepted for publication in Plant Direct.

Please view the editors' and reviewers' comments below and use their suggestions as a guide while you work on your revision.

When uploading the revised version of this article, please be sure to include the following:

-A word document that contains your response to the reviewers. You should respond to each reviewer comment and note the changes made to the manuscript. If you do not agree with a reviewer's comment and choose not to make a suggested revision, please explain why. Please try to provide as complete an answer as possible to each reviewer's criticisms.
-A tracked changes document with each change highlighted
- A clean version of the latest version of the manuscript

Plant Direct
Open Access

To upload your revision, please click the link below.
https://plantdirect.msubmit.net/cgi-bin/main.plex?el=A1Lr6gi4A3mGg2I7A9ftd2BCvpMCsRhr6DC30M1BwAZ

In order to provide as timely a service as possible, we ask that your revision is resubmitted within three months after receipt of this request. If an extension is needed, please send a request, along with a brief explanation, to the editorial office at plantdirect@wiley.com .

Please note that, in addition to publishing reviewer comments, the author's responses to review comments will also be published alongside the final version of the paper. If you would not like the author's responses to be published, please contact the editorial office at plantdirect@wiley.com .

Thank you very much for giving us an opportunity to review your work. I look forward to receiving the next version.

Sincerely,

Ana Fortes

Ana Fortes

Editor, Plant Direct

--- Editor comments:

--- Reviewer comments:
Reviewer #1:

The author investigated phenotypic variation in the disc diameter of sunflower seeds across different accessions over three years. The broad sense heritability of the trait was estimated to be 0.88. Genotyping by sequencing identified numerous high-quality SNPs, leading to a genome-wide association study that identified significant genetic loci associated with disc diameter, offering the potential for improving sunflower oil yield. The study has proposed a potential candidate gene responsible for controlling the size of sunflower seed discs, suggesting that these results could be valuable in enhancing sunflower cultivation practices.
Overall, the manuscript was well-written, and the experiments were conducted effectively, along with proficient statistical analysis.

Major concerns:
Since study primarily focuses on identifying candidate genes related to the disc diameter trait in sunflower seeds. I recommend that the authors provide a more detailed explanation of the identified candidate genes. In the discussion, the author can speculate on the potential involvement of these genes in the disc diameter trait by drawing insights from known functional genes.
Line 324-342: I suggest making a table for identified candidate genes instead of writing them in the text.

Reviewer #2:

In this study, Delen et al. performed a genome-wide association study in sunflower, revealing that the Chr10 locus can elevate the disc diameter by 4 cm, and the Chr16 locus increases by 3 cm. To understand the genetics of sunflowers concerning disc diameter, the authors genotyped a diverse set of sunflower accessions (N = 274) using a tunable genotyping-by-sequencing technique. Overall, the study seems well-planned, the article is well-written, the materials and methods are fine, and the results are beautifully explained with reasonable conclusions. However, I have some minor comments and suggestions and I would encourage the authors to consider them before proceeding to the next step.

Minor comments and suggestions:
L192-196: Please adjust the figures after their first citation in the text.
L256: Close the bracket.
L304-305: The authors have mentioned, "The Chr10 locus can elevate the disc diameter by 4

cm, and the Chr16 locus increases by 3 cm. Please elaborate on the effects of Chr on variation in the disc size.
L307: Please replace "marker assistant" with "marker-assisted".
L311: Write "2" as "Two".
L342: Add "and" before "proline-rich protein 36-like (LOC110919168)"
L342: Remove "were identified" or consider rephrasing the sentence.
L360: Most of the references are old. I would suggest supporting your objectives, hypothesis, and findings with the studies published in the recent past. Add some articles from 2024.

Decision Letter Round 2:

September 22, 2024
Prof. Jinliang Yang
University of Nebraska-Lincoln
363 Keim Hall
Lincoln, Nebraska 68583

MSID: 2024-01451R1
MS TITLE: Dissecting the genetic architecture of sunflower disc diameter using genome-wide association study

Dear Dr. Jinliang Yang:

I am pleased to inform you that your manuscript "Dissecting the genetic architecture of sunflower disc diameter using genome-wide association study" has been accepted for publication in Plant Direct. Thank you for utilizing our transfer feature, which I hope was a convenient option to share your work.

Your article will appear online in the next available issue of Plant Direct. To ensure your article gets published as quickly as possible, please pay attention to the steps detailed below. We have found that most of the delays happen at this stage, especially at the payment stage, so please respond as quickly as possible when prompted.

License Agreement: Once your article has been accepted it will move to Production and undergo admin and file checking - you may receive an email with any queries we have at this stage. When all required items are received by the publisher and queries resolved, the corresponding author will receive an email from Wiley's Author Services system which will ask

them to log on at https://authorservices.wiley.com/bauthor and will present them with the appropriate license for completion. Your article cannot be published until both the signed license agreement and payment of the article fee have been received.

Payment of the Open Access Article Publication Fee: All articles published in Plant Direct are fully open access: immediately and freely available to read, download and share. Plant Direct charges a publication fee to cover the publication costs. The corresponding author for this manuscript should have already received a quote from the payments team (cs-openaccess@wiley.com) with the estimated article publication fee; please email cs-openaccess@wiley.com if this has not been received. The corresponding author should log on to the Wiley Author Services site, where the publication fee can be paid by credit card or an invoice. Pro Forma can also be requested. Payment of the publication charge must be received before the article will be published online.

Proofs: You will have the opportunity to look over your paper once more when you receive the author proofs for your article. The proofs will be with you in approximately two weeks. Please note that, in addition to publishing reviewer comments, the author's responses to review comments will also be published alongside the final version of the paper. If you would not like the author's responses to be published, please contact the editorial office at plantdirect@wiley.com .

Promotion of your article: You can help your research get the attention it deserves! Check out Wiley's free Promotion Guide for best-practice recommendations for promoting your work at www.wileyauthors.com/eeo/guide. And learn more about Wiley Editing Services which offers professional video, design, and writing services to create shareable video abstracts, infographics, conference posters, lay summaries, and research news stories for your research at www.wileyauthors.com/eeo/promotion.

Thank you again for your contribution to Plant Direct. If you have any questions, feel free to contact the editorial office at plantdirect@wiley.com .

Sincerely,

Larry York

Larry York

Plant Direct

Open Access

Editor, Plant Direct

-- Editor comments

-- Reviewer comments:

Response to Reviewer:

Reviewer #1:

The author investigated phenotypic variation in the disc diameter of sunflower seeds across different accessions over three years. The broad sense heritability of the trait was estimated to be 0.88. Genotyping by sequencing identified numerous high-quality SNPs, leading to a genome-wide association study that identified significant genetic loci associated with disc diameter, offering the potential for improving sunflower oil yield. The study has proposed a potential candidate gene responsible for controlling the size of sunflower seed discs, suggesting that these results could be valuable in enhancing sunflower cultivation practices.
Overall, the manuscript was well-written, and the experiments were conducted effectively, along with proficient statistical analysis.

Major concerns:
Since study primarily focuses on identifying candidate genes related to the disc diameter trait in sunflower seeds. I recommend that the authors provide a more detailed explanation of the identified candidate genes. In the discussion, the author can speculate on the potential involvement of these genes in the disc diameter trait by drawing insights from known functional genes.

Response:

Thanks for the suggestions. As recommended, we extended the discussion section to include the potential involvement of the candidate genes and their possible effects on disc diameter. See lines 327-372 in the revised manuscript.

Line 324-342: I suggest making a table for identified candidate genes instead of writing them in the text.

Response:

We are grateful for this suggestion. For the Line 324-342, a table was prepared and given as Supplementary Table S7.

Reviewer #2:

In this study, Delen et al. performed a genome-wide association study in sunflower, revealing that the Chr10 locus can elevate the disc diameter by 4 cm, and the Chr16 locus increases by 3 cm. To understand the genetics of sunflowers concerning disc diameter, the authors genotyped a diverse set of sunflower accessions (N = 274) using a tunable genotyping-by-sequencing technique. Overall, the study seems well-planned, the article is well-written, the materials and methods are fine, and the results are beautifully explained with reasonable conclusions. However, I have some minor comments and suggestions and I would encourage the authors to consider them before proceeding to the next step.

Minor comments and suggestions:
L192-196: Please adjust the figures after their first citation in the text.

Response:

Thanks for your valuable suggestion. The figure was replaced after first citation (Line198-203).

L256: Close the bracket.

Response:

Thanks for catching the typo. We closed the bracket in the revised manuscript.

L304-305: The authors have mentioned, "The Chr10 locus can elevate the disc diameter by 4 cm, and the Chr16 locus increases by 3 cm. Please elaborate on the effects of Chr on variation in the disc size.

Response:

Thanks for the suggestion. As Reviewer 1 suggested, we have added some discussion on the candidate genes underlying the GWAS peaks and provided our speculation on the potential gene functions affecting disc diameter.

L307: Please replace "marker assistant" with "marker-assisted".

Response:

Thanks. It was replaced as per the reviewer's suggestiom.

L311: Write "2" as "Two".

Response:

It was changed as recommended.

L342: Add "and" before "proline-rich protein 36-like (LOC110919168)"

Response:

Thanks for the recommendation. In the revised manuscript, this part was removed and the genes mentioned were listed in a table (Supplementary table S9).

L342: Remove "were identified" or consider rephrasing the sentence.

Response:

Thanks for the suggestion. Again, this part has been removed in the revised manuscript.

L360: Most of the references are old. I would suggest supporting your objectives, hypothesis, and findings with the studies published in the recent past. Add some articles from 2024.

Response:

We appreciate the reviewer's comments and suggestions. We have added a number of recent publications using comparative genomics and GWAS in sunflower in the discussion section.
